# Avaliação da Velocidade da Onda de Pulso por Meio de Ecocardiografia Transtorácica em Crianças com Válvula Aórtica Bicúspide

**DOI:** 10.36660/abc.20250560

**Published:** 2026-05-06

**Authors:** Kerem Ertaş, Özlem Gül

**Affiliations:** 1 Department of Pediatric Cardiology Diyarbakir Children’s Hospital Diyarbakır Turquia Department of Pediatric Cardiology - Diyarbakir Children’s Hospital, Diyarbakır – Turquia

**Keywords:** Doença da Válvula Aórtica Bicúspide, Criança, Análise de Onda de Pulso

## Abstract

**Fundamento:**

O aumento da rigidez aórtica em pacientes com válvula aórtica bicúspide (VAB) pode afetar significativamente a morbidade e a mortalidade.

**Objetivo:**

Nosso objetivo foi avaliar as propriedades de elasticidade da aorta e a usabilidade da nova técnica, medindo a velocidade da onda de pulso (VOP) com um dispositivo de ecocardiografia transtorácica em pacientes com VAB.

**Métodos:**

Cinquenta pacientes com VAB e 50 crianças saudáveis com características demográficas semelhantes foram incluídos neste estudo. Os pacientes com VAB foram agrupados de acordo com a morfologia das cúspides fundidas. A função valvar era normal ou quase normal nos pacientes com VAB incluídos no estudo. A função ventricular esquerda, o diâmetro da raiz da aorta e as características de elasticidade da aorta de todos os pacientes foram avaliadas. Um valor p <0,05 foi considerado significativo.

**Resultados:**

A VOP foi significativamente maior no grupo com VAB (p=0,000). Os parâmetros de elasticidade aórtica obtidos a partir dos diâmetros da aorta ascendente foram semelhantes entre os grupos. Os diâmetros da aorta ascendente foram significativamente maiores no grupo com VAB. Houve correlação entre a VOP, o diâmetro da aorta ascendente, a vena contracta na regurgitação aórtica e a velocidade de pico aórtica. Os valores alfa de confiabilidade interobservador e intraobservador da VOP foram de 0,92 e 0,84, respectivamente.

**Conclusões:**

No grupo pediátrico com VAB e função valvar normal ou quase normal, embora os parâmetros de elasticidade aórtica fossem normais com as medidas convencionais baseadas nos diâmetros da aorta ascendente, as medidas de VOP com o mesmo dispositivo foram significativamente maiores, sem a necessidade de um aparelho ou programa adicional, e a rigidez aórtica aumentou no grupo com VAB. A medição da VOP por ecocardiografia é uma técnica confiável e reprodutível.

## Graphical abstract

**Figura Central:**
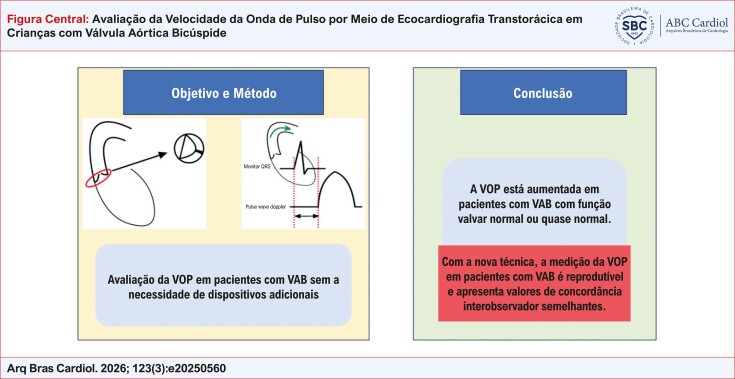
Avaliação da Velocidade da Onda de Pulso por Meio de Ecocardiografia Transtorácica em Crianças com Válvula Aórtica Bicúspide

A medição da velocidade da onda de pulso por ecocardiografia transtorácica é prática, de fácil acesso, de baixo custo, não requer dispositivos adicionais e pode fornecer informações importantes sobre a elasticidade da aorta, mesmo em estágios iniciais, em comparação com as medições convencionais. A medição da velocidade da onda de pulso com a nova técnica apresenta concordância interobservador semelhante. VOP: velocidade da onda de pulso; VAB: válvula aórtica bicúspide

## Introdução

A válvula aórtica bicúspide (VAB) é a cardiopatia congênita mais comum. A incidência na população geral é de aproximadamente 1-2%.^1^

A VAB ocorre em diferentes graus de fusão valvar. Como resultado dessa fusão, se desenvolvem patologias como estenose e insuficiência valvar. As propriedades elásticas da aorta se alteram na VAB devido aos efeitos hemodinâmicos da válvula e à diminuição da elastina na parede aórtica.^2^

Diversos métodos de imagem têm sido utilizados para avaliar as propriedades de elasticidade da aorta.^3-9^ As medições convencionais dos parâmetros de elasticidade da aorta por ecocardiografia transtorácica se baseiam nas medidas dos diâmetros sistólico e diastólico da aorta ascendente na vista longitudinal do ventrículo esquerdo. Essas medidas são obtidas a partir dos diâmetros anteroposteriores da aorta. No entanto, na valva aórtica bicúspide (VAB), a geometria da aorta ascendente varia devido à anatomia das cúspides fundidas.^10^ Portanto, as medições baseadas no princípio da variação do diâmetro podem não refletir a situação real. A velocidade da onda de pulso (VOP), o padrão ouro para a medição da elasticidade da aorta, apresenta limitações, como a necessidade de equipamentos especializados e tempo, a necessidade de pessoal treinado e o fato de que a distância entre os vasos pode não refletir a distância real entre eles devido à medição na pele.^11-13^ A VOP é a razão entre a distância entre os vasos determinados e a diferença de tempo no fluxo sanguíneo para atingir esses vasos.^11^ Em outras palavras, fornece uma ideia da velocidade do fluxo sanguíneo nos vasos. A elasticidade aórtica comprometida naturalmente aumenta a velocidade do fluxo. Em nosso estudo, objetivamos avaliar as propriedades de condução do fluxo sanguíneo local na aorta, medindo a distância entre as aortas ascendente e descendente e dividindo o tempo decorrido entre a chegada do sangue a cada uma delas pela diferença de tempo. Dessa forma, buscamos medir a VOP local na aorta sem a necessidade de softwares ou equipamentos especializados e avaliar a confiabilidade desse método de medição.

## Métodos

### População do estudo

Cinquenta pacientes com VAB e 50 crianças saudáveis com características demográficas semelhantes foram incluídos no estudo. Os pacientes saudáveis eram aqueles que compareceram ao ambulatório de cardiologia pediátrica com quaisquer queixas, e nenhuma patologia cardíaca foi detectada como resultado das avaliações.

Antes de todos os pacientes serem avaliados pelo mesmo cardiologista pediátrico, foram medidos o peso, a altura e a pressão arterial sistólica e diastólica (PAS, PAD). A relação entre o peso e a altura ao quadrado foi calculada como índice de massa corporal (IMC).^14^

O software G*power foi usado para calcular um tamanho de amostra de 50 participantes com um erro do Tipo I de 5%, poder de 85% e um tamanho de efeito de 0,61 (Heinrich-Heine-Universität Düsseldorf, Renânia do Norte-Vestfália, Alemanha).

Pacientes com VAB com velocidade aórtica máxima abaixo de 3 m/s e relação diâmetro de regurgitação aórtica/área de superfície corporal (ASC), índice de vena contracta (IVC) de 2 mm/m^2^ foram incluídos no estudo.^15,16^ A velocidade aórtica máxima foi medida nas pontas da válvula aórtica.

Este é um estudo prospectivo. O consentimento livre e esclarecido por escrito foi obtido de todos os pacientes participantes do estudo, e a aprovação foi concedida pelo Comitê de Ética do Hospital de Ensino e Pesquisa Gazi Yaşargil de Diyarbakır (parecer nº 530/2023).

A avaliação ecocardiográfica foi realizada de forma cega. As medições da VOP foram realizadas por dois observadores (1 e 2). As avaliações feitas pelo Observador 1 em momentos diferentes foram definidas como intraobservador, e as avaliações feitas pelos Observadores 1 e 2 foram definidas como interobservador.

### Ecocardiografia

Todos os pacientes foram avaliados pelo mesmo cardiologista pediátrico, foi realizada anamnese e exame físico detalhado. O ecocardiograma foi realizado em todos os pacientes na posição de decúbito lateral esquerdo (Vivid S60, 9; General Electric Healthcare, GE Vingmed, Noruega). Utilizando um transdutor de 1,5-4 MHz, foram obtidas imagens apicais de quatro, duas e três câmaras, além de imagens paraesternais de eixo longo e curto para avaliação dos modos 2D, colorido e M, e os registros foram coletados.^17^Foram medidas a espessura da parede do ventrículo esquerdo, o diâmetro do ventrículo esquerdo, a função sistólica e os achados da ecocardiografia em modo M no eixo longo do ventrículo esquerdo.

Ao posicionar o cursor do Doppler pulsado (PW) na ponta da válvula mitral na posição apical de 4 câmaras, foram medidas a velocidade E, a velocidade A, a relação E/A e o tempo de desaceleração (TD). As velocidades teciduais iniciais e tardias (e e a, respectivamente) e a velocidade tecidual sistólica foram medidas na parede lateral da valva mitral na vista apical de quatro câmaras.^17^

#### Classificação da morfologia da válvula aórtica

**Figura 1:**
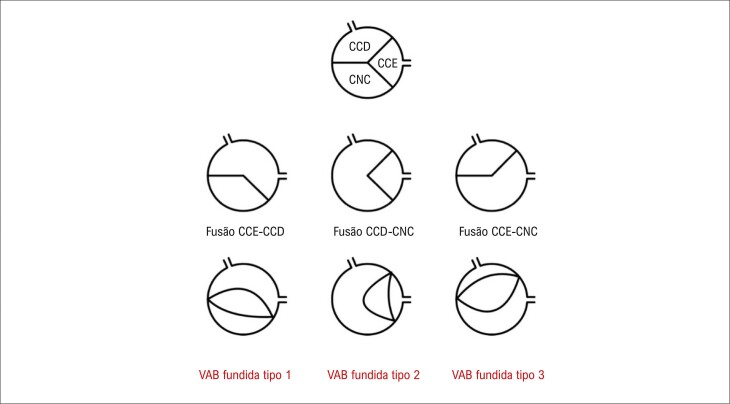
– Morfologias da válvula aórtica bicúspide. CCE: cúspide coronária esquerda; CCD: cúspide coronária direita; CNC: cúspide não coronária; VAB: válvula aórtica bicúspide

A valva aórtica bicúspide foi definida de acordo com a abertura da válvula aórtica durante a sístole, observada em imagens paraesternais de eixo curto. Existem muitas classificações de acordo com a classificação da VAB. A forma com duas ou três cúspides e duas cúspides funcionais é classificada como VAB fusionada; a forma com duas cúspides e morfologia quase simétrica é classificada como VAB do tipo 2-seio; e a forma com três cúspides e três cúspides funcionais é classificada como tipo de fusão parcial. A VAB fusionada, forma em que há duas ou três cúspides e duas cúspides funcionais como resultado da fusão, é classificada de acordo com a morfologia. Assim, a fusão da cúspide coronária esquerda (CCE) com a cúspide coronária direita (CCD) é classificada como tipo 1, formada pela CCD, e a fusão da cúspide não coronária (CNC) é classificada como tipo 2. O tipo formado pela fusão CCE-CNC é classificado como VAB fusionada tipo 3. Nenhum dos pacientes apresentou VAB fusionada tipo 3 em nosso estudo (Figura 1).^10^

#### Avaliação dos diâmetros da aorta

Os diâmetros do anel aórtico (AAo), do seio de Valsalva (SV) e da junção sinotubular (JST) foram medidos em meados da sístole.^17^ A dilatação da aorta foi definida como um escore z >2 em pelo menos uma localização do diâmetro da raiz da aorta ou da aorta ascendente.^18^

#### Avaliação da elasticidade da aorta

**Figura 2:**
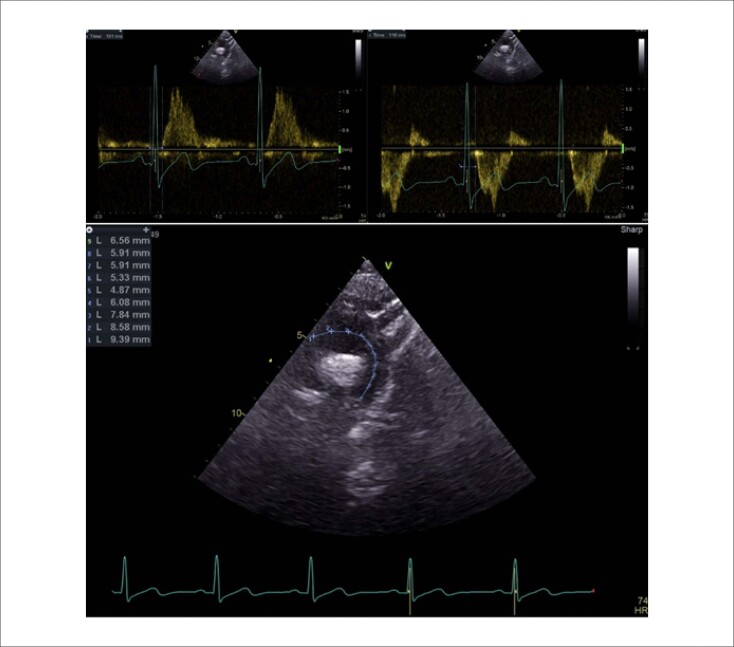
– Cálculo dos tempos desde a onda QRS até o traçado do fluxo na aorta (ascendente e descendente) no eletrocardiograma monitorado em um paciente com válvula aórtica bicúspide, e medição da distância entre os locais onde o fluxo é medido na aorta ascendente e descendente. Tempo medido a partir da aorta ascendente (superior esquerdo), tempo medido a partir da aorta descendente (superior direito), medição da distância entre a aorta ascendente e descendente (inferior).

A velocidade foi medida ao nível da válvula aórtica utilizando PW na vista supraesternal. Amostras de fluxo foram coletadas das aortas ascendente e descendente centrais utilizando PW. No eletrocardiograma (ECG) simultâneo, calculou-se o tempo decorrido entre o pico da onda QRS e o início do traçado de fluxo. A diferença entre os tempos medidos na aorta descendente e na aorta ascendente é denominada tempo de trânsito. A distância foi medida a partir dos pontos do cursor do PW nas aortas ascendente e descendente. As medições foram feitas a partir do centro da aorta, paralelamente às paredes do vaso. A relação entre essa distância e o tempo de trânsito é chamada de VOP e é calculada medindo a distância entre os pontos de medição na aorta ascendente e na aorta descendente (Figura 2).^19,20^ O tempo necessário para atingir as aortas ascendente e descendente foi medido a uma velocidade de varredura de 100 mm/s. O fluxo turbulento na aorta ocorre em casos como estenose e dilatação aórtica, o que causa diferentes perfis de fluxo em diferentes partes do vaso. Portanto, o volume de amostragem no lúmen aórtico foi definido em 100 mm/s para aumentar a precisão da temporização.^21^

**Figura 3:**
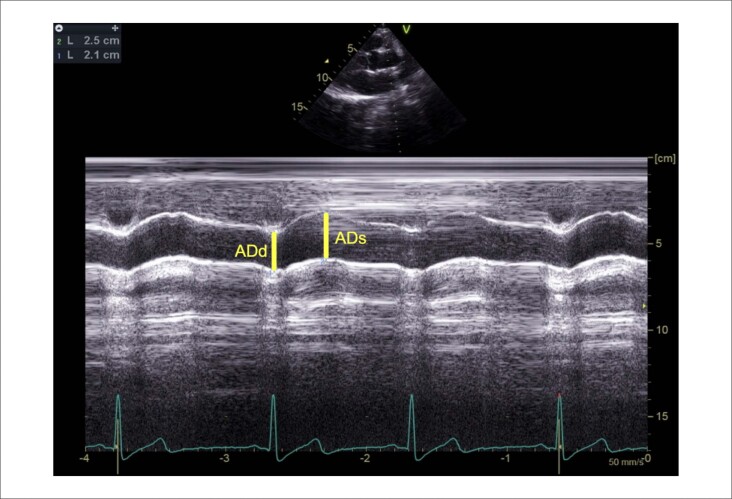
– Medição dos diâmetros aórticos sistólico e diastólico. ADs: diâmetro aórtico sistólico; ADd: diâmetro aórtico diastólico.

Os diâmetros aórticos sistólico e diastólico (DAs e DAd, respectivamente) foram calculados usando o modo M ao longo do eixo longitudinal da aorta ascendente. As fórmulas de deformação aórtica, índice de rigidez aórtica e distensibilidade aórtica foram calculadas a partir desses diâmetros (Figura 3).

- Deformação aórtica $\left(\mathrm{DA},\%)=100^{*}[(\mathrm{DAs}-\mathrm{DAd})/\mathrm{DAd}\right]$ ,
- Índice de rigidez (IR)= logaritmo natural(PAS/PAD)/[DA] ,
- A distensibilidade (D)=2×[DA/ pressão de pulso] é calculada.^9^

## Análise estatística

O software SPSS (SPSS, Chicago, versão 27) foi utilizado para a análise estatística dos dados. As variáveis contínuas são expressas como média ± desvio padrão ou mediana (percentis 25 e 75).intervalo interquartil, IIQ), e as variáveis categóricas são expressas em porcentagens. O teste de Kolmogorov-Smirnov foi utilizado para avaliar a distribuição normal. O teste-t de Student independente ou o teste de Mann-Whitney foram utilizados dependendo da distribuição normal das variáveis contínuas, e o teste qui-quadrado foi utilizado para a análise de variáveis categóricas. Os testes de Pearson e Spearman foram utilizados para a análise de correlação de acordo com a homogeneidade da distribuição dos parâmetros.

Os valores de variabilidade interobservador e intraobservador foram obtidos avaliando variáveis contínuas por dois observadores e pelo mesmo observador em momentos diferentes, respectivamente. A análise de Bland-Altman foi utilizada para avaliar a concordância entre as medições.^22^ Foram calculados a diferença média, o desvio padrão (DP) e o limite de concordância (LC) entre as medições. A fórmula [(média)±(1,96∗ desvio padrão) ] foi usada para calcular o intervalo do LC.^23^ A análise de regressão linear foi utilizada para avaliar e modelar as relações entre as variáveis. O teste-t foi utilizado para determinar o intervalo de confiança (IC) de 95%. A confiabilidade foi avaliada pela análise dos valores de alfa de Cronbach. Um alfa de Cronbach > 0,7 foi considerado significativo.^24^

A significância estatística foi definida como p < 0,05.

## Resultados

Os valores de idade, peso, altura, ASC, IMC, PAS, pressão arterial diastólica (PAD) e pressão de pulso (PP) foram semelhantes em ambos os grupos, sem diferenças estatísticas detectadas. 72% dos pacientes com VAB e 73% dos pacientes do grupo controle eram do sexo masculino.

Nenhum procedimento cirúrgico ou intervencionista foi realizado em nenhum dos pacientes com VAB. Não foram observadas anomalias extracardíacas. Setenta e dois por cento dos pacientes com VAB apresentavam VAB fusionada tipo 1 e 28% apresentavam VAB fusionada tipo 2.

A regurgitação aórtica estava presente em 44% dos pacientes com VAB. No grupo com VAB e regurgitação aórtica, 72% apresentavam VAB tipo 1 e 28% VAB tipo 2 fusionada. A dilatação aórtica estava presente em 16% dos pacientes com VAB.

O diâmetro diastólico final do ventrículo esquerdo, a fração de ejeção do ventrículo esquerdo e o encurtamento fracional do ventrículo esquerdo foram semelhantes em ambos os grupos, e nenhuma diferença estatística foi detectada.

As velocidades E e A do ventrículo esquerdo, a relação E/A e os valores de TD foram semelhantes em ambos os grupos, e nenhuma diferença estatística foi detectada.

As velocidades teciduais sistólicas e diastólicas da parede lateral da valva mitral e a relação E/e foram semelhantes (Tabela 1).

**Tabela 1:** – Tabela comparativa dos parâmetros demográficos e ecocardiográficos entre os grupos

Parâmetro	Grupo VAB (n=50)	Grupo de controle (n=50)	Valor-p
Sexo (M/F,%)	72/28	73/27	1,00^‡^
Idade (anos)	8,33 (5,31-12,81)	8,66 (5,56-13,52)	0,77^†^
Peso (kg)	31,00 (± 16,91)	34,65 (± 15,50)	0,37^*^
Altura (cm)	133,52 (±25,39)	137,76 (±24,68)	0,54^*^
ASC (m^2^)	1,04 (± 0,38)	1,13 (± 0,35)	0,36^*^
IMC (kg/m^2^)	16,08 (± 2,97)	17,29 (± 2,71)	0,09^*^
PAS (mmHg)	108,76 (±13,58)	110,07 (±14,52)	0,74^*^
PAD (mmHg)	61,96 (±8,53)	61,88 (±10,40)	0,98^*^
PP (mmHg)	45,00 (33,00-71,00)	44,50 (30,00-75,00)	0,74^†^
FE (%)	70,00 (±5,83)	70,19 (±6,78)	0,91^*^
EF (%)	39,25 (±4,47)	39,38 (±5,61)	0,92^*^
DDVE (mm)	40,91 (±7,36)	39,60 (±6,96)	0,52^*^
E (m/s)	1,07 (1,01-1,24)	1,01 (0,94-1,14)	0,06^†^
A (m/s)	0,70 (±0,10)	0,64 (±0,14)	0,14^*^
Relação E/A	1,63 (±0,26)	1,68 (±0,40)	0,58^*^
TD (ms)	123,12 (±28,48)	120,53 (±15,94)	0,69^*^
Velocidade mitral e (m/s)	0,19 (± 0,03)	0,19 (± 0,03)	0,81^*^
Velocidade mitral a (m/s)	0,08 (0,06-12)	0,08 (0,06-0,12)	0,86^†^
Velocidade mitral s (m/s)	0,09 (0,07-0,16)	0,10 (0,08-0,13)	0,28^†^
E/e	6,05 (± 1,16)	5,54 (± 1,21)	0,52^*^

*: teste-t de Student; ^†^Teste de Mann-Whitney; ^‡^Teste do qui-quadrado; ASC: área de superfície corporal; IMC: índice de massa corporal; PAS: pressão arterial sistólica; PAD: pressão arterial diastólica; DDVE: diâmetro diastólico final do ventrículo esquerdo; FE: fração de ejeção; EF: Encurtamento fracional; E: velocidade diastólica inicial do fluxo mitral; A: velocidade diastólica tardia do fluxo mitral; TD: tempo de desaceleração; e: velocidade diastólica inicial do Doppler tecidual mitral; a: velocidade diastólica tardia do Doppler tecidual mitral; s: velocidade sistólica do Doppler tecidual mitral

A velocidade aórtica máxima foi significativamente maior no grupo com VAB do que no grupo controle (p=0,003).

Os diâmetros do DAs, DAd, AAo, SV e JST foram significativamente maiores no grupo VAB (valores de p 0,003, 0,005, 0,000, 0,000 e 0,000, respectivamente).

A deformação aórtica, a distensibilidade e o índice de rigidez foram semelhantes em ambos os grupos, sem diferenças estatísticas detectadas.

A VOP foi significativamente maior no grupo VAB do que no grupo controle (p=0,000) (Tabela 2).

**Tabela 2:** – Comparação da velocidade aórtica máxima, diâmetro aórtico, parâmetros de elasticidade aórtica, tempo para atingir a aorta ascendente e descendente, tempo de trânsito e distância entre a aorta ascendente e descendente nos grupos com válvula aórtica bicúspide e controle

Parâmetro	Grupo VAB (n=50)	Grupo de controle (n=50)	Valor-p
Velocidade aórtica máxima (m/s)	1,46 (± 0,35)	1,20 (± 0,22)	0,003^*^
IVC (mm/m^2^)	1,55 (± 0,57)	-	-
DAs (mm)	21,54 (± 4,92)	17,94 (± 3,10)	0,003^*^
Dad (mm)	18:00 (13-30)	16 (10-19)	0,005^†^
AAo (mm)	18,57 (± 3,51)	14,45 (± 2,70)	0,000^*^
SV (mm)	24,22 (± 4,90)	18,55 (± 3,50)	0,000^*^
JST (mm)	20,92 (± 3,94)	14,83 (± 2,70)	0,000^*^
Deformação aórtica (%)	19,90 (± 8,30)	18,27 (± 8,48)	0,07^*^
Índice de rigidez	4,85 (2,95-10,93)	3,42 (2,33-4,96)	0,07^†^
Distensibilidade (10-6.cm^2^.dina^-1^)	0,004 (0,002-0,009)	0,007 (0,005-0,010)	0,07^†^
VOP (m/s)	4,86 (3,50-7,56)	2,78 (2,00-3,29)	0,000^†^
Tempo para atingir AA (ms)	88,46 (± 14,84)	82,83 (± 13,25)	0,13^*^
Tempo para atingir AD (ms)	101,25 (± 13,61)	105,55 (± 13,98)	0,24^*^
Tempo de trânsito (ms)	11,50 (9:00-17:00)	21:00 (17:00-30:00)	0,000^†^
Distância entre AA e AD (mm)	57,06 (46,63-68,79)	56,88 (47,93-64,81)	0,88^†^

*: teste t de Student; ^†^: Teste de Mann-Whitney; AA: aorta ascendente; AD: aorta descendente; AAo: anel aórtico; SV: seio de Valsalva; JST: junção sinotubular da aorta; DAs: diâmetro aórtico sistólico; DAd: diâmetro aórtico diastólico; VOP: velocidade da onda de pulso; IVC: índice da vena contracta

Embora o tempo para atingir a aorta ascendente e descendente e a distância entre a aorta ascendente e descendente fossem semelhantes em ambos os grupos, o tempo de trânsito foi significativamente menor no grupo VAB (p=0,000).

Ao comparar os VAB fundidos tipo 1 e tipo 2, os valores de idade, sexo, ASC, IMC, diâmetros da aorta e parâmetros de elasticidade da aorta foram semelhantes em ambos os grupos (Tabela 3).

**Tabela 3:** – Comparação de parâmetros demográficos, diâmetros da aorta, velocidade aórtica máxima, índice de vena contracta de insuficiência aórtica, parâmetros de elasticidade da aorta, tempo para atingir a aorta ascendente e descendente, tempo de trânsito e distância entre a aorta ascendente e descendente em fenótipos de válvula aórtica bicúspide

Parâmetros	VAB fundida tipo 1 (n=36)	VAB fundida tipo 2 (n=14)	Valor-p
Idade (anos)	10.04 (6.37-13.08)	6,66 (4,91-13,00)	0,60^†^
Sexo (M/F, %)	86/14	71/29	0,96^‡^
ASC (m^2^)	1,11 (± 0,41)	0,96 (± 0,39)	0,42^*^
IMC (kg/m^2^)	16,46 (± 3,02)	15,89 (± 3,20)	0,68^*^
DAs (mm)	22,14 (± 5,00)	20,00 (± 4,72)	0,34^*^
Dad (mm)	19,61 (± 4,64)	17,42 (± 4,23)	0,29^*^
AAo (mm)	18,21 (± 3,49)	20,52 (± 4,13)	0,57^*^
SV (mm)	24,38 (± 5,52)	23,33 (± 3,74)	0,79^*^
JST (mm)	20,84 (± 4,19)	21,34 (± 3,52)	0,88^*^
Velocidade aórtica máxima (m/s)	1,47 (± 0,37)	1,43 (± 0,30)	0,79^*^
IVC (mm/m^2^)	1,56 (± 0,52)	1,52 (± 0,82)	0,93^*^
Deformação aórtica (%)	13,44 (± 8,46)	15,09 (± 8,40)	0,66^*^
Índice de rigidez (IR)	4,41 (3,20-12,20)	4,45 (2,70-6,61)	0,46^†^
Distensibilidade (10-6 cm^2^ dina^-1^)	0,005 (0,002-0,008)	0,004 (0,005-0,010)	0,50^†^
VOP (m/s)	5,03 (± 2,01)	3,77 (± 1,11)	0,13^*^
Tempo para atingir AA (ms)	89,64 (± 15,35)	83,42 (± 9,21)	0,33^*^
Tempo para atingir AD (ms)	102,47 (± 13,56)	99,85 (± 9,90)	0,65^*^
Tempo de trânsito (ms)	12,82 (± 4,42)	16,42 (± 7,13)	0,14^*^
Distância entre AA e AD (mm)	56,95 (± 11,44)	59,90 (± 20,62)	0,88^*^

*: teste-t de Student; ^†^: Teste de Mann-Whitney; ^‡^: Teste do qui-quadrado; ASC: área de superfície corporal; IMC: índice de massa corporal;; AAo: anel aórtico; SV: seio de Valsalva aórtico; JST: junção sinotubular; DAs: diâmetro aórtico sistólico; DAd: diâmetro aórtico diastólico; VOP: velocidade da onda de pulso; AA: aorta ascendente; Ad: aorta descendente; IVC: índice da vena contracta

As correlações entre a VOP e o diâmetro da aorta são mostradas na Tabela 4. Foi encontrada uma correlação positiva entre a VOP e os diâmetros da raiz da aorta, a velocidade aórtica de pico, a vena contracta na regurgitação aórtica e o tempo para atingir a aorta descendente; nenhuma correlação foi encontrada entre a VOP e o tempo para atingir a aorta descendente, a deformação aórtica, a distensibilidade ou o índice de rigidez. Houve uma correlação negativa entre a VOP e o tempo de trânsito. Houve uma correlação entre a vena contracta da regurgitação aórtica e os diâmetros da aorta. Nenhuma correlação foi encontrada entre a velocidade aórtica de pico e os diâmetros da aorta. Em nosso estudo, o aumento da VOP em pacientes VAB foi visualizado em uma ilustração central com um dispositivo de ecocardiografia (Figura Central).

**Tabela 4:** – Correlações entre vários parâmetros

Parâmetros		Valor- r	Valor-p
VOP	Anel aórtico	0,27	0,10^§^
	Seio de Valsalva	0,25	0,13^§^
	JST	0,36	0,009^§^
	DAs	0,38	0,005^§^
	DAd	0,39	0,004^§^
	Deformação aórtica	-0,11	0,42^§^
	Rigidez aórtica	0,15	0,30^§^
	Distensibilidade aórtica	-0,17	0,21^§^
	Velocidade aórtica máxima	0,30	0,03^§^
	RA vena contracta	0,43	0,001^§^
	Tempo para atingir AA	0,47	0,000^§^
	Tempo para atingir AD	0,009	0,95^§^
	Tempo de trânsito	-0,83	0,000^§^
RA vena contracta	DAs	0,58	0,000^§^
	DAd	0,52	0,007^§^
Velocidade aórtica máxima	DAs	0,22	0,12^//^

^§^Teste de Spearman; ^//^: Teste de Pearson; SJT: junção sinotubular da aorta; Das: diâmetro aórtico sistólico; Dad: diâmetro aórtico diastólico; RA: regurgitação aórtica; AA: aorta ascendente; AD: aorta descendente

As diferenças médias, DPs, LCs e valores de confiabilidade da variabilidade intra e interobservador estão resumidos na Tabela 5. Os valores alfa de confiabilidade inter e intraobservador da VOP foram 0,84 e 0,92, respectivamente. Os valores alfa de confiabilidade inter e intraobservador da distância foram 0,78 e 0,99, respectivamente. Os valores alfa de confiabilidade inter e intraobservador do tempo de trânsito foram 0,86 e 0,93.

**Tabela 5:** – Tabela com os seguintes parâmetros: velocidade da onda de pulso, distância, tempo de trânsito, valores de diferença da variabilidade inter e intraobservador, média, desvio padrão, intervalo de valores superior e inferior do limite de concordância, intervalo de confiança de 95%, alfa (α) e valor-p

	Velocidade da onda de pulso (VOP) (m/s)						
	n	Valor da diferença			IC de 95%	Confiabilidade	
		Média (m/s)	DP	Intervalo LC		Alfa (α)	Valor- p
Variabilidade interobservadores	50	0,19	0,54	-0,66, 1,06	0,72, 0,92	0,84	0,000
Variabilidade intraobservador	50	0,14	0,40	-0,50, 0,79	0,86, 0,96	0,92	0,000
	**Distância (mm)**						
	**n**	**Valor da diferença**			**IC de 95%**	**Confiabilidade**	
		**Média (mm)**	**DP**	**Intervalo LC**		**Alfa (α)**	**Valor- p**
Variabilidade interobservadores	50	2,36	7.23	-9,47, 14,18	0,60, 0,88	0,78	0,004
Variabilidade intraobservador	50	-1,60	1,51	6,18, 2,97	0,98, 0,99	0,99	0,000
	**Tempo de trânsito (ms)**						
	**n**	**Valor da diferença**			**IC de 95%**	**Confiabilidade**	
		**Média (ms)**	**DP**	**Intervalo LC**		**Alfa (α)**	**Valor- p**
Variabilidade interobservadores	50	-0,06	2.07	-4,19, 4,06	0,75, 0,92	0,86	0,000
Variabilidade intraobservador	50	-1,20	2,00	-6,31, 3,91	0,87, 0,96	0,93	0,000

IC: intervalo de confiança; LC: limite de concordância; mm: milímetro; ms: milissegundo; m: metro; s: segundo; DP: desvio padrão.

**Figura 5:**
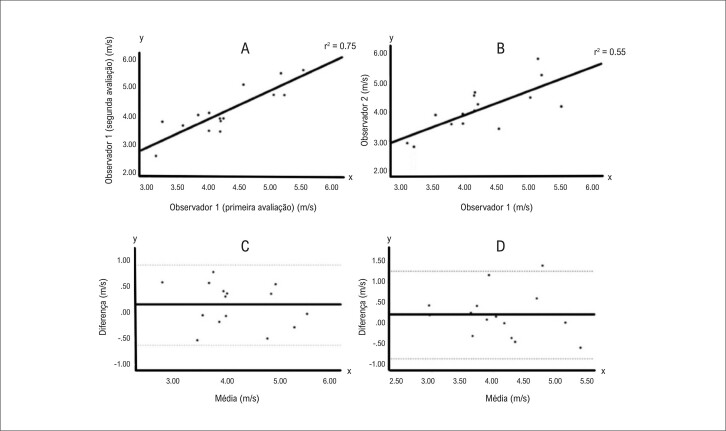
– Gráficos de correlação intraobservador (A) e interobservador (B) da velocidade da onda de pulso, gráficos de Bland-Altman intraobservador (C) e interobservador (D).

Os gráficos de Bland-Altman intra e interobservador dos valores de VOP e o gráfico de correlação entre as observações são mostrados na Figura 5.

## Discussão

O resultado mais básico do nosso estudo é que em pacientes com VAB com função ventricular preservada e função valvar normal ou quase normal, a VOP medida por ecocardiografia — sem a necessidade de software adicional — mostra um aumento, indicativo de rigidez aórtica elevada. Acreditamos que a elasticidade da aorta está comprometida em pacientes com VAB, mesmo na primeira infância, e que as medidas convencionais não refletem a realidade devido às diferentes geometrias aórticas nesses pacientes. Acreditamos que até mesmo pequenas alterações na função da válvula afetam a geometria da aorta devido à alteração do fluxo sanguíneo. Acreditamos que o novo método que utilizamos é importante porque é prático, reproduzível e fornece informações precoces sobre a função elástica da aorta.

O sistema arterial, tanto em estrutura quanto em função, consiste em grandes artérias elásticas e artérias musculares. Os grandes vasos elásticos armazenam o sangue ejetado durante a sístole ventricular devido às fibras elásticas que contêm.^25^ Durante esse período, quase metade do sangue ejetado atinge as artérias musculares. Posteriormente, quando a válvula aórtica se fecha e o ciclo diastólico se inicia, as artérias musculares mantêm a pressão e o fluxo sanguíneo contínuo pelo sistema vascular. A aorta torácica é composta por 40% de fibras elásticas.^26,27^ A VAB é uma das cardiopatias congênitas mais comuns. Pode ocorrer isoladamente ou acompanhada por outras síndromes ou anomalias. (1) A VAB é formada embriologicamente pela fusão da válvula aórtica em graus variáveis. Juntamente com as alterações hemodinâmicas que ocorrem como resultado da estenose e insuficiência valvar, a disfunção endotelial e a degeneração das fibras elásticas da aorta ocorrem na VAB.^28^ Consequentemente, as propriedades elásticas da aorta são alteradas em pacientes com VAB. O aumento da rigidez arterial em doenças cardiovasculares afeta significativamente a morbidade e a mortalidade.^29,30^ Considerando a fisiologia dos vasos ao longo do ciclo cardíaco, a deterioração das propriedades elásticas da aorta pode causar problemas de perfusão em muitos órgãos.

Na avaliação da elasticidade da aorta, são utilizados métodos como ecocardiografia, tomografia computadorizada ou ressonância magnética, que avaliam alterações no diâmetro ou na área da secção transversal da aorta ascendente, bem como exame Doppler tecidual da aorta, avaliação da espessura íntima-média da carótida e medições invasivas. Vários métodos, incluindo a medição de velocidade, foram usados para este propósito.^3-9,20^ Esses métodos apresentam diversas limitações, como a necessidade de pessoal treinado, requisitos de tempo e custo, problemas técnicos e exposição à anestesia e materiais de contraste.^12,31-33^

A VOP é o padrão ouro para a avaliação da rigidez arterial.^4^ A VOP pode ser medida tanto de forma invasiva quanto não invasiva. As medições invasivas e não invasivas da VOP produzem resultados semelhantes.^20^ O fato de a VOP ser afetada pela viscosidade sanguínea é uma limitação da VOP aórtica.^34^ Existem algumas desvantagens na avaliação da VOP usando medidas ultrassonográficas. Principalmente, as medidas foram obtidas das artérias periféricas. A distância entre as duas artérias periféricas foi medida a partir da pele. A distância medida a partir da pele, devido à angulação e ao trajeto dos vasos, é uma desvantagem dessa técnica.^13^ Além disso, na medição com essa técnica, o tempo, os requisitos de dispositivos especiais, equipamentos específicos e o uso de contraste em medições invasivas são limitações dessa técnica.^11,12^

**Figura 4:**
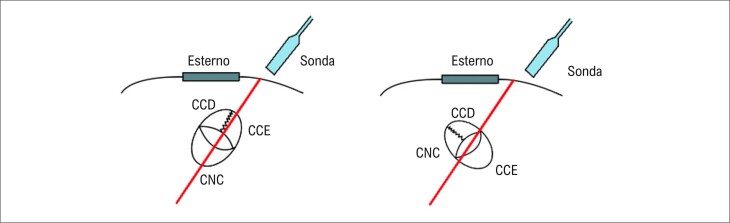
– Orientação da sonda em relação ao eixo da aorta de acordo com os fenótipos da válvula aórtica bicúspide. À esquerda, o eixo longitudinal da VAB fundida tipo 1 é perpendicular ao ângulo da sonda, enquanto à direita, o eixo transversal da aorta na VAB fundida tipo 2 é perpendicular ao diâmetro da sonda. CCE: cúspide coronária esquerda; CCD: cúspide coronária direita; CNC: cúspide não coronária.

Convencionalmente, as medidas de elasticidade da aorta por ecocardiografia se baseiam nas alterações do diâmetro durante a sístole e a diástole em cortes do modo M da aorta ascendente.^17^ No entanto, hipotetizamos que essas medidas seriam pouco confiáveis na VAB devido às alterações na estrutura geométrica da raiz da aorta. Além disso, como a pressão arterial é medida de forma não invasiva na periferia, ela pode não refletir os parâmetros de distensibilidade ou rigidez da aorta. Portanto, nosso objetivo foi avaliar a precisão das medidas da VOP por ecocardiografia transtorácica sem equipamentos ou softwares adicionais. Com base no princípio de que a distância percorrida por unidade de tempo é o percurso, calculamos o valor local da VOP pela razão entre a distância entre os pontos de medição na aorta ascendente e descendente e a diferença no tempo de percurso do sangue até a aorta ascendente e descendente. Em nosso estudo, apesar da dilatação aórtica no grupo com VAB, os parâmetros de elasticidade obtidos pelo método convencional não diferiram entre os grupos. Resultados semelhantes aos nossos em um estudo corroboram essa hipótese.^35^ Na VAB, o eixo do segmento fundido é longo, enquanto o eixo perpendicular ao segmento fundido é mais curto. Na VAB tipo 1, o eixo longitudinal da aorta é paralelo à sonda de ecocardiografia, enquanto no tipo 2, é perpendicular à sonda (Figura 4). Em nosso estudo, o diâmetro da aorta ascendente no grupo com VAB tipo 1 foi maior do que no grupo com VAB tipo 2, embora a diferença não tenha sido estatisticamente significativa, o que reforça ainda mais essa hipótese.

Em nosso estudo, as funções sistólica e diastólica ventriculares foram normais no grupo com VAB. Os pacientes com VAB avaliados foram selecionados dentre aqueles com função valvar normal ou quase normal. Na prática clínica, a PP aumenta quando a elasticidade da aorta está comprometida. Os valores normais de pressão arterial e PP observados em todos os grupos do nosso estudo podem estar relacionados ao fato de que a elasticidade aórtica comprometida ainda não causou disfunção ventricular ou de outros órgãos. Entretanto, é necessário um acompanhamento a longo prazo para confirmar essa hipótese.

A elasticidade intacta da aorta pode não se refletir nos sistemas arteriais periféricos. Portanto, a avaliação da rigidez arterial periférica pode não indicar a elasticidade da aorta. Estudos indicaram que a rigidez aórtica é a primeira a ser afetada pela rigidez arterial, com a rigidez periférica se desenvolvendo posteriormente.^29,30^

O mecanismo de dilatação da aorta na VAB pode ser o comprometimento do estresse da parede devido à insuficiência da valva aórtica ou à turbulência que ocorre na estenose da valva aórtica.^36,37^O risco de dilatação da aorta também foi relatado como aumentado independentemente da função valvar.^38^ A dilatação da raiz da aorta foi relatada entre 15,1% e 39,1% em pacientes com VAB isolada.^39^ No presente estudo, a incidência de dilatação da raiz da aorta foi de 16%. Como os pacientes do grupo VAB em nosso estudo foram selecionados com função valvar normal ou quase normal, a avaliação da incidência de dilatação da raiz da aorta não fornecerá resultados precisos. A VAB é um fator de risco independente para dissecção da aorta.^40^

Estudos relatam que a VOP é alta ou normal em pacientes com VAB.^3,41-45^ O aumento da VOP pode prejudicar a função aórtica na determinação da distribuição sanguínea.^26^ Isso pode levar a doenças cardiovasculares.^46^ Os valores alfa de confiabilidade intraobservador e interobservador da VOP foram de 0,92 e 0,84, respectivamente. Os altos valores intraobservador e interobservador indicam a confiabilidade e reprodutibilidade da técnica de medição.^47^ Em nosso estudo, a diferença nos valores médios e de desvio padrão da VOP foi de 0,19 e 0,54 m/s entre os observadores, respectivamente. Isso demonstra excelente concordância de acordo com as diretrizes da *ARTERY Society*.^48^ Embora nosso método de medição tenha sido diferente, acreditamos que esses valores possam ser significativos. Uma limitação do nosso estudo é que não comparamos as medições da VOP obtidas por métodos ultrassonográficos com aquelas obtidas por ecocardiografia. Acreditamos que a praticidade da técnica de medição da VOP sem a necessidade de um aparelho extra, sua reprodutibilidade e sua alta confiabilidade fornecerão informações importantes sobre o acompanhamento e o prognóstico dos pacientes.

### Limitações

O fato de o estudo ter sido realizado em um único centro e apresentar um número reduzido de pacientes foram as principais limitações. Outra limitação foi o número de pacientes no grupo com valva aórtica bicúspide tipo 2, inferior ao do grupo com valva aórtica bicúspide tipo 1. Na medição da VOP, a distância entre as aortas ascendente e descendente foi medida intermitentemente utilizando o dispositivo atual. Os erros de medição também representaram uma limitação deste estudo. O valor alfa de confiabilidade interobservador foi de 0,78, o mais baixo já observado, e a medição ininterrupta da distância inclinada no dispositivo pode eliminar essa desvantagem. Outra limitação é a influência da viscosidade sanguínea na VOP. Além disso, não foi realizado um estudo de viabilidade das técnicas de medição da VOP. Uma limitação do nosso estudo foi a ausência de comparação entre as medições da VOP obtidas por métodos ultrassonográficos e aquelas obtidas por ecocardiografia.

## Conclusões

O aumento da rigidez aórtica em pacientes com VAB foi demonstrado pelo valor da VOP com o mesmo dispositivo de ecocardiografia, sem a necessidade de um dispositivo adicional. Embora sejam normais quando avaliados com parâmetros de elasticidade derivados dos diâmetros aórticos por ecocardiografia, a elasticidade aórtica está reduzida mesmo no período inicial quando avaliada pela VOP. A avaliação de pacientes com VAB com parâmetros de elasticidade aórtica medidos convencionalmente por ecocardiografia pode gerar resultados incorretos. Acreditamos que a medição da VOP com um dispositivo de ecocardiografia, a avaliação e o acompanhamento em pacientes com VAB podem fornecer informações mais úteis. Em pacientes com VAB, mesmo uma doença valvar aórtica mínima, particularmente a regurgitação aórtica, pode causar deterioração da elasticidade aórtica. Acreditamos que, no acompanhamento de pacientes com VAB, especialmente na presença de regurgitação aórtica, a avaliação com VOP, em adição ao método convencional de avaliação das propriedades de elasticidade aórtica, fornecerá informações mais precisas. A elevada confiabilidade intraobservador e interobservador da VOP medida por ecocardiografia transtorácica demonstra que se trata de uma técnica clinicamente reprodutível e confiável.

## References

[1] Schaefer BM, Lewin MB, Stout KK, Gill E, Prueitt A, Byers PH (2008). The Bicuspid Aortic Valve: An Integrated Phenotypic Classification of Leaflet Morphology and Aortic Root Shape. Heart.

[2] Antequera-González B, Martínez-Micaelo N, Alegret JM (2020). Bicuspid Aortic Valve and Endothelial Dysfunction: Current Evidence and Potential Therapeutic Targets. Front Physiol.

[3] Boonyasirinant T, Rajiah P, Flamm SD (2019). Abnormal Aortic Stiffness in Patients with Bicuspid Aortic Valve: Phenotypic Variation Determined by Magnetic Resonance Imaging. Int J Cardiovasc Imaging.

[4] Covic A, Siriopol D (2015). Pulse Wave Velocity Ratio: The New "Gold Standard" for Measuring Arterial Stiffness. Hypertension.

[5] Ganten M, Boese JM, Leitermann D, Semmler W (2005). Quantification of Aortic Elasticity: Development and Experimental Validation of a Method Using Computed Tomography. Eur Radiol.

[6] Gürses D, Ozyürek AR, Levent E, Ulger Z (2012). Elastic Properties of the Abdominal Aorta in the Children with Bicuspid Aortic Valve: An Observational Study. Anadolu Kardiyol Derg.

[7] Oren A, Vos LE, Uiterwaal CS, Grobbee DE, Bots ML (2003). Aortic Stiffness and Carotid Intima-Media Thickness: Two Independent Markers of Subclinical Vascular Damage in Young Adults?. Eur J Clin Invest.

[8] Pac FA, Guray Y, Polat TB (2010). Wall Motion Velocities of Ascending Aorta Measured by Tissue Doppler Imaging in Obese Children. Pediatr Int.

[9] Stefanadis C, Stratos C, Boudoulas H, Kourouklis C, Toutouzas P (1990). Distensibility of the Ascending Aorta: Comparison of Invasive And Non-Invasive Techniques in Healthy Men and in Men with Coronary Artery Disease. Eur Heart J.

[10] Michelena HI, Della Corte A, Evangelista A, Maleszewski JJ, Edwards WD, Roman MJ (2021). International Consensus Statement on Nomenclature and Classification of the Congenital Bicuspid Aortic Valve and Its Aortopathy, for Clinical, Surgical, Interventional and Research Purposes. Eur J Cardiothorac Surg.

[11] Allen J (2007). Photoplethysmography and its Application in Clinical Physiological Measurement. Physiol Meas.

[12] Pereira T, Correia C, Cardoso J (2015). Novel Methods for Pulse Wave Velocity Measurement. J Med Biol Eng.

[13] Tillin T, Chambers J, Malik I, Coady E, Byrd S, Mayet J (2007). Measurement of Pulse Wave Velocity: Site Matters. J Hypertens.

[14] Guo SS, Chumlea WC (1999). Tracking of Body Mass Index in Children in Relation to Overweight in Adulthood. Am J Clin Nutr.

[15] Colan SD, Sleeper LA (2020). Longitudinal Variation in Presence and Severity of Cardiac Valve Regurgitation in Healthy Children. J Am Soc Echocardiogr.

[16] Oulego-Erroz I, Alonso-Quintela P, Mora-Matilla M, Minaya SG, Armentia SLL (2013). Ascending Aorta Elasticity in Children with Isolated Bicuspid Aortic Valve. Int J Cardiol.

[17] Lopez L, Saurers DL, Barker PCA, Cohen MS, Colan SD, Dwyer J (2024). Guidelines for Performing a Comprehensive Pediatric Transthoracic Echocardiogram: Recommendations from the American Society of Echocardiography. J Am Soc Echocardiogr.

[18] Savis A, Haseler E, Beardsley H, Chowienczyk PJ, Simpson JM, Sinha MD (2024). Aortic Dilatation in Children and Young People with ADPKD. Kidney Int Rep.

[19] Sandor GG, Hishitani T, Petty RE, Potts MT, Desouza A, Desouza E (2003). A novel Doppler Echocardiographic Method of Measuring the Biophysical Properties of the Aorta in Pediatric Patients. J Am Soc Echocardiogr.

[20] Styczynski G, Rdzanek A, Pietrasik A, Kochman J, Huczek Z, Sobieraj P (2016). Echocardiographic Assessment of Aortic Pulse-Wave Velocity: Validation Against Invasive Pressure Measurements. J Am Soc Echocardiogr.

[21] Jo CO, Lande MB, Meagher CC, Wang H, Vermilion RP (2010). A Simple Method of Measuring Thoracic Aortic Pulse Wave Velocity in Children: Methods and Normal Values. J Am Soc Echocardiogr.

[22] Bland JM, Altman DG (1986). Statistical Methods for Assessing Agreement between Two Methods of Clinical Measurement. Lancet.

[23] Swinscow TDV, Campbell MJ (1997). Statistics at Square One.

[24] Bujang MA, Omar ED, Baharum NA (2018). A Review on Sample Size Determination for Cronbach's Alpha Test: A Simple Guide for Researchers. Malays J Med Sci.

[25] Belz GG (1995). Elastic Properties and Windkessel Function of the Human Aorta. Cardiovasc Drugs Ther.

[26] Gkaliagkousi E, Douma S (2009). The Pathogenesis of Arterial Stiffness and Its Prognostic Value in Essential Hypertension and Cardiovascular Diseases. Hippokratia.

[27] Mileva N, Velikova T, Velikov T, Vassilev D (2024). Aortic Elasticity and Cardiovascular Risk Stratification: A Narrative Review on the Current Understanding. J Vasc Dis.

[28] Yassine NM, Shahram JT, Body SC (2017). Pathogenic Mechanisms of Bicuspid Aortic Valve Aortopathy. Front Physiol.

[29] Laurent S, Boutouyrie P, Lacolley P (2005). Structural and Genetic Bases of Arterial Stiffness. Hypertension.

[30] Mitchell GF, Parise H, Benjamin EJ, Larson MG, Keyes MJ (2004). Changes in Arterial Stiffness and Wave Reflection with Advancing Age in Healthy Men and Women: The Framingham Heart Study. Hypertension.

[31] Drozdz J, Erbel R, Zamorano J, Erbel R, Zamorano J (1995). Atlas of Tissue Doppler Echocardiography - TDE.

[32] Pilz N, Heinz V, Ax T, Fesseler L, Patzak A, Bothe TL (2024). Pulse Wave Velocity: Methodology, Clinical Applications, and Interplay with Heart Rate Variability. Rev Cardiovasc Med.

[33] Rusk RA, Li XN, Irvine T, Mori Y, Wanitkun S, Li XK (2002). Surface Integration of Velocity Vectors from 3D Digital Colour Doppler: An Angle Independent Method for Laminar Flow Measurements. Eur J Echocardiogr.

[34] Kim JY, Yoon J, Cho M, Lee BK, Karimi A, Shin S (2013). Blood Characteristics Effect on Pulse Wave Velocity. Clin Hemorheol Microcirc.

[35] Pees C, Michel-Behnke I (2012). Morphology of the Bicuspid Aortic Valve and Elasticity of the Adjacent Aorta in Children. Am J Cardiol.

[36] Thanassoulis G, Yip JW, Filion K, Jamorski M, Webb G, Siu SC (2008). Retrospective Study to Identify Predictors of the Presence and Rapid Progression of Aortic Dilatation in Patients with Bicuspid Aortic Valves. Nat Clin Pract Cardiovasc Med.

[37] Ward C (2000). Clinical Significance of the Bicuspid Aortic Valve. Heart.

[38] Verma S, Siu SC (2014). Aortic Dilatation in Patients with Bicuspid Aortic Valve. N Engl J Med.

[39] Spaziani G, Ballo P, Favilli S, Fibbi V, Buonincontri L, Pollini I (2014). Clinical Outcome, Valve Dysfunction, and Progressive Aortic Dilation in a Pediatric Population with Isolated Bicuspid Aortic Valve. Pediatr Cardiol.

[40] Nistri S, Grande-Allen J, Noale M, Basso C, Siviero P, Maggi S (2008). Aortic Elasticity and Size in Bicuspid Aortic Valve Syndrome. Eur Heart J.

[41] Kosger P, Akin T, Kiztanir H, Ucar B (2021). Arterial Stiffness and Left Ventricular Myocardial Function in Children with a Well-Functioning Bicuspid Aortic Valve. Arq Bras Cardiol.

[42] Warner PJ, Al-Quthami A, Brooks EL, Kelley-Hedgepeth A, Patvardhan E, Kuvin JT (2013). Augmentation index and Aortic Stiffness in Bicuspid Aortic Valve Patients with Non-Dilated Proximal Aortas. BMC Cardiovasc Disord.

[43] Ekici F, Uslu D, Bozkurt S (2017). Elasticity of Ascending Aorta and Left Ventricular Myocardial Functions in Children with Bicuspid Aortic Valve. Echocardiography.

[44] Erolu E, Akalin F, Çetiner N, Saylan BÇ (2018). Aortic Elasticity and the Influence of Valve Morphology in Children with Bicuspid Aortic Valve. Cardiol Young.

[45] Weismann CG, Lombardi KC, Grell BS, Northrup V, Sugeng L (2016). Aortic Stiffness and Left Ventricular Diastolic Function in Children with Well-Functioning Bicuspid Aortic Valves. Eur Heart J Cardiovasc Imaging.

[46] Agabiti-Rosei E, Mancia G, O'Rourke MF, Roman MJ, Safar ME, Smulyan H (2007). Central Blood Pressure Measurements and Antihypertensive Therapy: A Consensus Document. Hypertension.

[47] Campo D, Khettab H, Yu R, Genain N, Edouard P, Buard N (2017). Measurement of Aortic Pulse Wave Velocity with a Connected Bathroom Scale. Am J Hypertens.

[48] Spronck B, Terentes-Printzios D, Avolio AP, Boutouyrie P, Guala A, Jeroncic A (2024). 2024 Recommendations for Validation of Noninvasive Arterial Pulse Wave Velocity Measurement Devices. Hypertension.

