# Supplementary material for: Avaliação da Velocidade da Onda de Pulso por Meio de Ecocardiografia Transtorácica em Crianças com Válvula Aórtica Bicúspide
Source: Arq Bras Cardiol. 2026 May 6;123(3):e20250560. [Article in Portuguese] doi: 10.36660/abc.20250560 (PMC13398813; doi:10.36660/abc.20250560)
Supplement: Translation

# Translation: Evaluation of Pulse Wave Velocity using Transthoracic Echocardiography in Children with Bicuspid Aortic Valve

**DOI:** 10.36660/abc.20250560i

**Authors:** Kerem Ertaş, Özlem Gül

**Affiliations:** 1 Türkiye Department of Pediatric Cardiology - Diyarbakir Children’s Hospital, Diyarbakır – Türkiye

**Keywords:** Bicuspid Aortic Valve Disease, Child, Pulse Wave Analysis

## Abstract

**Background:**

Increased aortic stiffness in patients with bicuspid aortic valve (BAV) may significantly affect morbidity and mortality.

**Objective:**

We aimed to evaluate aortic elasticity properties and the usability of the new technique by measuring pulse wave velocity (PWV) with a transthoracic echocardiography device in patients with BAV.

**Methods:**

Fifty patients with BAV and 50 healthy children with similar demographic characteristics were included in this study. Patients with BAV were grouped according to fused cusp morphology. Valve function was normal or near normal in the patients with BAV included in the study. All patients’ left ventricular function, aortic root diameter, and aortic elasticity characteristics were evaluated. A p-value <0.05 was considered significant.

**Results:**

PWV was significantly higher in the BAV group (p=0.000). Aortic elasticity parameters obtained from ascending aorta diameters were similar between the groups. Ascending aorta diameters were significantly higher in the BAV group. There was a correlation between PWV, ascending aortic diameter, vena contracta in aortic regurgitation, and peak aortic velocity. PWV inter- and intraobserver reliability alpha values were 0.92 and 0.84, respectively.

**Conclusions:**

In the pediatric BAV group with normal or near-normal valve function, although aortic elasticity parameters were normal with conventional measurements based on ascending aorta diameters, PWV measurements with the same device were significantly higher without the need for an additional apparatus or program, and aortic stiffness increased in the BAV group. PWV measurement by echocardiography is a reliable and reproducible technique.

## Introduction

The bicuspid aortic valve (BAV) is the most common congenital heart disease. The incidence in the general population is approximately 1-2%.^1^

BAV occurs at varying degrees of valve fusion. As a result of valve fusion, pathologies such as valve stenosis and valve insufficiency develop. Aortic elasticity properties change in BAV due to the hemodynamic effects of the valve and decreased elastin in the aortic wall.^2^

Many imaging methods have been used to evaluate aortic elasticity properties.^3-9^ Conventional measurements of aortic elasticity parameters using transthoracic echocardiography are based on measurements of the systolic and diastolic diameters of the ascending aorta in the longitudinal view of the left ventricle. These measurements were obtained from the anteroposterior diameters of the aorta. However, in BAV, the geometry of the ascending aorta varies because of the fused cusp anatomy.^10^ Therefore, measurements based on the principle of diameter variation may not reflect the actual situation. Pulse wave velocity (PWV), the gold standard for aortic elasticity measurement, has limitations, such as the need for specialized equipment and time, the need for trained personnel, and the fact that the distance between vessels may not reflect the actual distance between vessels due to the skin measurement.^11-13^ PWV is the ratio of the distance between the determined vessels to the time difference in blood reaching these vessels.^11^ In other words, it provides an idea of how fast the vessels are flowing. Impaired aortic elasticity naturally increases the flow velocity. In our study, we aimed to assess the local flow conduction properties of the aorta by measuring the distance between the ascending and descending aortas and dividing the time between blood reaching the ascending and descending aortas by the time difference. Thus, we aimed to measure aortic local PWV without requiring any specialized software or equipment and to assess the reliability of this measurement method.

## Methods

### Study population

Fifty patients with BAV and 50 healthy children with similar demographic characteristics were included in the study. Healthy patients consisted of those who visited the pediatric cardiology outpatient clinic with any complaints, and no cardiac pathology was detected as a result of the evaluations.

Before all patients were evaluated by the same pediatric cardiologist, weight, height, and systolic and diastolic blood pressure (SBP, DBP) were measured. The ratio of weight to height in square meters was calculated as body mass index (BMI).^14^

G*power software was used to calculate a sample size of 50 participants with a 5% Type I error, 85% power, and an effect size of 0.61 (Heinrich-Heine-Universität Düsseldorf, North Rhine-Westphalia, Germany).

BAV patients with peak aortic velocity below 3m/s and aortic regurgitation diameter to body surface area (BSA) ratio, vena contracta index of 2mm/m^2^ were included in the study.^15,16^ Peak aortic velocity was measured at the aortic valve tips.

This is a prospective study. Written informed consent was obtained from all patients in the study, and approval was obtained from the Diyarbakır Gazi Yaşargil Training and Research Hospital Ethics Committee (decision no.530/2023).

Echocardiographic evaluation was performed blindly. PWV measurements were performed by two observers (1 and 2). The evaluations made by Observer 1 at different times were defined as intraobserver, and the evaluations made by Observers 1 and 2 were defined as interobserver.

### Echocardiography

All patients were evaluated by the same pediatric cardiologist, anamnesis was performed, and a detailed physical examination was performed. Echocardiography was performed in all patients in the left lateral decubitus position (Vivid S60, 9; General Electric Healthcare, GE Vingmed, Norway). Using a 1.5-4 MHz transducer, apical four-, two-, and three-chamber, parasternal long-axis and short-axis images were used to evaluate 2D, color, and M-modes, and records were taken.^17^Left ventricular wall thickness, left ventricular diameter, systolic function, and left ventricular long-axis M-mode echocardiography findings were measured.

By placing the PW Doppler cursor on the mitral valve tip in the apical 4-chamber position, E velocity, A velocity, E/A ratio, and deceleration time (DT) were measured. Early and late tissue velocities (e and a, respectively) and systolic tissue velocity were measured from the mitral lateral wall in the apical 4-chamber view.^17^

### Classification of aortic valve morphology

BAV was defined according to the opening of the aortic valve in systole on parasternal short-axis imaging. There are many classifications according to the BAV classification. The form with two or three cusps and two functional cusps is a fused BAV; the form with two cusps and almost symmetrical morphology is a 2-sinus type BAV; and the form with three cusps and three functional cusps is classified as the partial fusion type. Fused BAV, the form where there are two or three cusps and two functional cusps as a result of fusion, is classified according to morphology. Accordingly, the left coronary cusp (LCC)-right coronary cusp (RCC) fusion is classified as type 1, formed by the RCC, and the non-coronary cusp (NCC) fusion is classified as type 2. The type formed by LCC-NCC fusion is classified as type 3 fused BAV. None of the patients had type 3 fused BAV in our study (Figure 1).^10^

**Figure 1:** – Bicuspid aortic valve morphologies. LCC: left coronary cusp; RCC: right coronary cusp; NCC: noncoronary cusp; BAV: bicuspid aortic valve.

### Assessment of aortic diameters

The aortic annulus (AAo), sinus of Valsalva (SV), and sinotubular junction (STJ) diameters were measured mid-systolically.^17^ Aortic dilatation was defined as a z-score of >2 at least one location of the aortic root diameter or ascending aorta.^18^

### Assessment of aortic elasticity

Velocity was measured at the aortic valve level using PW Doppler in the suprasternal view. Flow samples were taken from the central ascending and descending aortas using PW Doppler. In simultaneous electrocardiography, the time from the peak of the QRS wave to the region where the flow trace began was calculated. The difference between the times measured in the descending aorta and the ascending aorta is referred to as the transit time. Distance was measured from the PW cursor points of the ascending and descending aortas. Measurements were taken from the center of the aorta parallel to the vessel walls. The ratio of this distance to the transit time is called the PWV and is calculated by measuring the distance between the measured locations in the ascending aorta and the descending aorta (Figure 2).^19,20^ The time taken to reach the ascending and descending aortas was measured at a sweep speed of 100 mm/s. Turbulent flow in the aorta occurs in cases such as aortic stenosis and dilatation, and this causes different flow profiles in different parts of the vessel. Therefore, the sampling volume in the aortic lumen was set to 100 mm/s to increase timing accuracy.^21^

**Figure 2:** – Calculation of times from the QRS wave to the flow trace in the aorta (ascending and descending) on monitor electrocardiography in a patient with a bicuspid aortic valve, and measurement of the distance between the locations where flow is measured in the ascending and descending aorta. Time measured from ascending aorta (top left), time measured from descending aorta (top right), measurement of distance between ascending and descending aorta (bottom).

Systolic and diastolic aortic diameters (ADs and ADd, respectively) were calculated using the M-mode along the long axis of the ascending aorta. The aortic strain, aortic stiffness index, and aortic distensibility formulas were calculated from these diameters (Figure 3).

**Figure 3:** – Measurement of systolic and diastolic aortic diameters. ADs: systolic aortic diameter; ADd: diastolic aortic diameter.

- Aortic strain (AS,%)=100∗[(ADs−ADd)/ADd] ,
- Stiffness index (SI)=natural logarithm(SBP/DBP)/[AS] ,
- Distensibility (D)=2×[AS/ pulse pressure] is calculated.^9^

### Statistical Analysis

SPSS (SPSS, Chicago, version 27) software was used for the statistical analysis of the data. Continuous variables are expressed as mean±standard deviation or median (percentile 25 and 75, interquartile range, IQR), and categorical variables are expressed as percentages. The Kolmogorov-Smirnov test was used to evaluate the normal distribution. Independent Student’s T-test or Mann-Whitney test was used depending on whether continuous variables were normally distributed, and the chi-square test was used for analysis of categorical variables. Pearson’s and Spearman’s tests were used for correlation analysis according to the homogeneity of the parameter distribution.

Inter-observer and intra-observer variability values were obtained by evaluating continuous variables by two observers and the same observer at different times, respectively. Bland-Altman analysis was used to evaluate the agreement between measurements.^22^ The mean difference, standard deviation (SD), and limit of agreement (LOA) values between measurements were measured. The formula [(mean)±(1.96∗ standard deviation) ] was used to calculate the LOA range.^23^ Linear regression analysis was used to evaluate and model the relationships between the variables. A t-test was used to determine the 95% confidence interval (CI). Reliability was evaluated by analyzing the Cronbach’s alpha values. Cronbach’s alpha >0.7 was considered significant.^24^

Statistical significance was set at p < 0.05.

## Results

Age, weight, height, BSA, BMI, SBP, DBP, and pulse pressure (PP) values were similar in both groups, with no statistical differences detected. 72% of the BAV patients and 73% of the control group patients were male.

No surgical or interventional procedures were performed in any of the patients with BAV. There were no extracardiac anomalies. Seventy-two percent of BAV patients had type 1 fused BAV, and 28% had type 2 fused BAV.

Aortic regurgitation was present in 44% of BAV patients. In the BAV group with aortic regurgitation, 72% had the type 1 and 28% had the type 2 fused BAV. Aortic dilatation was present in 16% of patients with BAV.

The left ventricular end-diastolic diameter (LVEDd), left ventricular ejection fraction (EF), and left ventricular fractional shortening (FS) were similar in both groups, and no statistical difference was detected.

The left ventricular E and A velocities, E/A ratio, and DT values were similar in both groups, and no statistical difference was detected.

The mitral lateral wall systolic and diastolic tissue velocities and E/e ratio were similar (Table 1).

**Table 1:** – Table comparing demographic and echocardiographic parameters across groups

Parameter	BAV group (n=50)	Control group (n=50)	Valor-p
Sex (M/F,%)	72/28	73/27	1.00^‡^
Age (year)	8.33 (5.31-12.81)	8.66 (5.56-13.52)	0.77^†^
Weight (kg)	31.00 (± 16.91)	34.65 (± 15.50)	0.37^*^
Height (cm)	133.52 (±25.39)	137.76 (±24.68)	0.54^*^
BSA (m^2^)	1.04 (± 0.38)	1.13 (± 0.35)	0.36^*^
BMI (kg/m^2^)	16.08 (± 2.97)	17.29 (± 2.71)	0.09^*^
SBP (mmHg)	108.76 (±13.58)	110.07 (±14.52)	0.74^*^
DBP (mmHg)	61.96 (±8.53)	61.88 (±10.40)	0.98^*^
PP (mmHg)	45.00 (33.00-71.00)	44.50 (30.00-75.00)	0.74^†^
EF (%)	70.00 (±5.83)	70.19 (±6.78)	0.91^*^
FS (%)	39.25 (±4.47)	39.38 (±5.61)	0.92^*^
LVEDd (mm)	40.91 (±7.36)	39.60 (±6.96)	0.52^*^
E (m/s)	1.07 (1.01-1.24)	1.01 (0.94-1.14)	0.06^†^
A (m/s)	0.70 (±0.10)	0.64 (±0.14)	0.14^*^
E/A ratio	1.63 (±0.26)	1.68 (±0.40)	0.58^*^
DT (ms)	123.12 (±28.48)	120.53 (±15.94)	0.69^*^
Mitral e velocity (m/s)	0.19 (± 0.03)	0.19 (± 0.03)	0.81^*^
Mitral a velocity (m/s)	0.08 (0.06-12)	0.08 (0.06-0.12)	0.86^†^
Mitral s velocity (m/s)	0.09 (0.07-0.16)	0.10 (0.08-0.13)	0.28^†^
E/e	6.05 (± 1.16)	5.54 (± 1.21)	0.52^*^

*: student-T test; ^†^: Mann-Whitney test; ^‡^: Chi-square test; BSA: body surface area; BAV: bicuspid aortic valve; BMI: body mass index; SBP: systolic blood pressure; DBP: diastolic blood pressure; LVEDd: left ventricular end-diastolic diameter; EF: ejection fraction; FS: fraction short; E: mitral inflow early diastolic velocity; A: mitral inflow late diastolic velocity; DT: deceleration time; e: mitral tissue doppler early diastolic velocity; a: mitral tissue doppler late diastolic velocity; s: mitral tissue doppler systolic velocity;

The peak aortic velocity was significantly higher in the BAV group than in the control group (p=0.003).

ADs, ADd, AAo, SV, and STJ diameters were significantly higher in the BAV group (p-values 0.003, 0.005, 0.000, 0.000, and 0.000, respectively).

The aortic strain, distensibility, and stiffness index were similar in both groups, with no statistical differences detected.

The PWV was significantly higher in the BAV group than in the control group (p=0.000) (Table 2).

**Table 2:** – Comparison of peak aortic velocity, aortic diameter, aortic elasticity parameters, time to reach the ascending and descending aorta, transit time, and distance between ascending and descending aorta values in the bicuspid aortic valve and control groups

Parameter	BAV group (n=50)	Control group (n=50)	p-value
Peak aortic velocity (m/s)	1.46 (± 0.35)	1.20 (± 0.22)	0.003^*^
VCI (mm/m^2^)	1.55 (± 0.57)	-	-
ADs (mm)	21.54 (± 4.92)	17.94 (± 3.10)	0.003^*^
ADd (mm)	18.00 (13-30)	16 (10-19)	0.005^†^
AAo (mm)	18.57 (± 3.51)	14.45 (± 2.70)	0.000^*^
SV (mm)	24.22 (± 4.90)	18.55 (± 3.50)	0.000^*^
STJ (mm)	20.92 (± 3.94)	14.83 (± 2.70)	0.000^*^
Aortic strain (%)	19.90 (± 8.30)	18.27 (± 8.48)	0.07^*^
Stiffness index	4.85 (2.95-10.93)	3.42 (2.33-4.96)	0.07^†^
Distensibility (10^-6^.cm^2^.dyne^-1^)	0.004 (0.002-0.009)	0.007 (0.005-0.010)	0.07^†^
PWV (m/s)	4.86 (3.50-7.56)	2.78 (2.00-3.29)	0.000^†^
Time to reach the AA (ms)	88.46 (± 14.84)	82.83 (± 13.25)	0.13^*^
Time to reach DA (ms)	101.25 (± 13.61)	105.55 (± 13.98)	0.24^*^
Transit time (ms)	11.50 (9.00-17.00)	21.00 (17.00-30.00)	0.000^†^
Distance between AA and DA (mm)	57.06 (46.63-68.79)	56.88 (47.93-64.81)	0.88^†^

*: student-T test; ^†^: Mann-Whitney test; BAV: bicuspid aortic valve; AA: ascending aorta; DA: descending aorta; AAo: aortic annulus; SV: sinus of valsalva; STJ: sinotubular junction; ADs: systolic aortic diameter; ADd: diastolic aortic diameter; PWV: pulse wave velocity; VCI: vena contracta index

While the time to reach the ascending and descending aorta and the distance between the ascending and descending aorta were similar in both groups, the transit time was significantly lower in the BAV group (p=0.000).

When the type 1 and type 2 fused BAV were compared, the values for age, sex, BSA, BMI, aortic diameters, and aortic elasticity parameters were similar in both groups (Table 3).

**Table 3:** – Comparison of demographic parameters, aortic diameters, peak aortic velocity, vena contracta index of aortic insufficiency, aortic elasticity parameters, time to reach the ascending and descending aorta, transit time, and distance between ascending and descending aorta in bicuspid aortic valve phenotypes

Parameters	Type 1 fused BAV (n=36)	Type 2 fused BAV (n=14)	p-value
Age (year)	10.04 (6.37-13.08)	6.66 (4.91-13.00)	0.60^†^
Sex (M/F, %)	86/14	71/29	0.96^‡^
BSA (m^2^)	1.11 (± 0.41)	0.96 (± 0.39)	0.42^*^
BMI (kg/m^2^)	16.46 (± 3.02)	15.89 (± 3.20)	0.68^*^
ADs (mm)	22.14 (± 5.00)	20.00 (± 4.72)	0.34^*^
ADd (mm)	19.61 (± 4.64)	17.42 (± 4.23)	0.29^*^
AAo (mm)	18.21 (± 3.49)	20.52 (± 4.13)	0.57^*^
SV (mm)	24.38 (± 5.52)	23.33 (± 3.74)	0.79^*^
STJ (mm)	20.84 (± 4.19)	21.34 (± 3.52)	0.88^*^
Peak aortic velocity (m/s)	1.47 (± 0.37)	1.43 (± 0.30)	0.79^*^
VCI (mm/m^2^)	1.56 (± 0.52)	1.52 (± 0.82)	0.93^*^
Aortic strain (%)	13.44 (± 8.46)	15.09 (± 8.40)	0.66^*^
Stiffness index (SI)	4.41 (3.20-12.20)	4.45 (2.70-6.61)	0.46^†^
Distensibility (10^-6^ cm^2^ dyne^-1^)	0.005 (0.002-0.008)	0.004 (0.005-0.010)	0.50^†^
PWV (m/s)	5.03 (± 2.01)	3.77 (± 1.11)	0.13^*^
Time to reach the AA (ms)	89.64 (± 15.35)	83.42 (± 9.21)	0.33^*^
Time to reach DA (ms)	102.47 (± 13.56)	99.85 (± 9.90)	0.65^*^
Transit time (ms)	12.82 (± 4.42)	16.42 (± 7.13)	0.14^*^
Distance between AA and DA (mm)	56.95 (± 11.44)	59.90 (± 20.62)	0.88^*^

*: student-T test; ^†^: Mann-Whitney test; ^‡^: Chi-square test; BSA: body surface area; BMI: body mass index; BAV: bicuspid aortic valve; AAo: aortic annulus; SV: aortic sinus of valsalva; STJ: sinotubular junction; ADs: systolic aortic diameter; ADd: diastolic aortic diameter; PWV: pulse wave velocity; AA: ascending aorta; DA: descending aorta; VCI: vena contracta index

The correlations between PWV and aortic diameter are shown in Table 4. A positive correlation was found between PWV and aortic root diameters, peak aortic velocity, vena contracta in aortic regurgitation, and time to reach the descending aorta; no correlation was found between PWV and time to reach the descending aorta, aortic strain, distensibility, or stiffness index. There was a negative correlation between PWV and transit time. There was a correlation between the vena contracta of aortic regurgitation and aortic diameters. No correlation was found between peak aortic velocity and aortic diameters. In our study, the increase in PWV in BAV patients was visualized in a central illustration with an echocardiography device (Central Illustration).

**Table 4:** – Correlations between various parameters

Parameters		r-value	p-value
PWV	Aortic anulus	0.27	0.10^§^
Sinus valsalva	0.25	0.13^§^
STJ	0.36	0.009^§^
ADs	0.38	0.005^§^
ADd	0.39	0.004^§^
Aortic strain	-0.11	0.42^§^
Aortic stiffness	0.15	0.30^§^
Aortic distensibility	-0.17	0.21^§^
Peak aortic velocity	0.30	0.03^§^
AR vena contracta	0.43	0.001^§^
Time to reach the AA	0.47	0.000^§^
Time to reach DA	0.009	0.95^§^
Transit time	-0.83	0.000^§^
AR vena contracta	ADs	0.58	0.000^§^
	ADd	0.52	0.007^§^
Peak aortic velocity	ADs	0.22	0.12^//^

^§^: Spearman test; ^//^: Pearson test; PWV: pulse wave velocity; STJ: sinotubular junction; ADs: systolic aortic diameter; ADd: diastolic aortic diameter; AR: aortic regurgitation; AA: ascending aorta; DA: descending aorta.

The intra- and inter-observer variability difference means, standard deviations (SD), limits of agreement (LOA), and reliability values are summarized in Table 5. PWV inter- and intra-observer reliability alpha values were 0.84 and 0.92, respectively. The distance inter- and intra-observer reliability alpha values were 0.78 and 0.99, respectively. Transit time inter- and intra-observer reliability alpha values were 0.86 and 0.93.

**Table 5:** – Pulse wave velocity, distance, transit time parameters, inter- and intraobserver variability difference values, mean, standard deviation, limit of agreement upper and lower value range, and 95% confidence interval, alpha (α), and p-value table

	PWV (m/s)						
	n	Value of difference			95% CI	Reliability	
		Mean (m/s)	SD	LOA range		Alpha (α)	p value
Interobserver variability	50	0.19	0.54	-0.66, 1.06	0.72, 0.92	0.84	0.000
Interobserver variability	50	0.14	0.40	-0.50, 0.79	0.86, 0.96	0.92	0.000
	**Distance (mm)**						
	**n**	**Value of difference**			**95% CI**	**Reliability**	
		**Mean (mm)**	**SD**	**LOA range**		**Alpha (α)**	**p value**
Interobserver variability	50	2.36	7.23	-9.47, 14.18	0.60, 0.88	0.78	0.004
Interobserver variability	50	-1.60	1.51	6.18, 2.97	0.98, 0.99	0.99	0.000
	**Transit time (ms)**						
	**n**	**Value of difference**			**95% CI**	**Reliability**	
		**Mean (ms)**	**SD**	**LOA range**		**Alpha (α)**	**p value**
Interobserver variability	50	-0.06	2.07	-4.19, 4.06	0.75, 0.92	0.86	0.000
Interobserver variability	50	-1.20	2.00	-6.31, 3.91	0.87,0.96	0.93	0.000

PWV: Pulse wave velocity; CI: confidence interval, LOA: limit of agreement, mm: millimeter, ms: millisecond, m: meter, s: second, SD: standard deviation.

The intra- and inter-observer Bland-Altman plots of the PWV values and the correlation plot between the observations are shown in Figure 5.

**Figure 5:** – Intra-observer (A) and inter-observer (B) correlation plots of pulse wave velocity, intra-observer (C), and inter-observer (D) Bland-Altman plots.

## Discussion

The most basic result of our study is that in BAV patients with preserved ventricular function and normal or near-normal valve function, PWV measured through echocardiography—without the need for additional software—shows an increase, indicative of elevated aortic stiffness. We believe that aortic elasticity is impaired in patients with BAV, even in early childhood, and that conventional measurements do not reflect reality due to the different aortic geometries in these patients. We believe that even slight changes in valve function affect aortic geometry due to altered aortic flow. We believe that the new method we used is important because it is usable, reproducible, and provides early-stage information about aortic elastic function.

The arterial system, both in structure and function, consists of large elastic arteries and muscular arteries. Large elastic vessels store blood ejected during ventricular systole owing to the elastic fibers they contain.^25^ During this time, almost half of the ejected blood reaches the muscular arteries. Later, when the aortic valve closes, and the diastolic cycle begins, the muscular arteries maintain pressure and maintain continuous blood flow through the vascular system. The thoracic aorta consists of 40% elastic fibers.^26, 27^ BAV is one of the most common congenital heart diseases. It may be isolated or accompanied by other syndromes or anomalies. (1) BAV is embryologically formed by the fusion of the aortic valve to varying degrees. Along with the hemodynamic changes that occur as a result of valve stenosis and insufficiency, endothelial dysfunction and degeneration of aortic elastin fibers occur in BAV.^28^ Consequently, aortic elasticity properties are altered in patients with BAV. Increased arterial stiffness in cardiovascular diseases significantly affects morbidity and mortality.^29,30^ Considering the physiology of vessels throughout the cardiac cycle, deterioration of the aortic elastic properties can cause perfusion problems in many organs.

In the evaluation of aortic elasticity, methods such as echocardiography, computed tomography, or magnetic resonance imaging, which assess changes in the diameter or cross-sectional area of the ascending aorta, as well as aortic tissue Doppler examination, carotid intima-media thickness assessment, and invasive measurements, are utilized. Several methods, including velocity measurement, have been used for this purpose.^3-9,20^ These methods have various limitations, such as the need for trained personnel, time and cost requirements, technical problems, and exposure to anesthesia and contrast materials.^12,31-33^

PWV is the gold standard for the evaluation of arterial stiffness.^4^ PWV can be measured both invasively and non-invasively. Non-invasive and invasive PWV measurements yield similar results.^20^The fact that PWV is affected by blood viscosity is a limitation of the aortic PWV.^34^ There are some drawbacks to evaluating PWV using sonographic measurements. Primarily, measurements were obtained from the peripheral arteries. The distance between the two peripheral arteries was measured from the skin. The distance measured from the skin owing to the angulation and course of the vessels is a disadvantage of this technique.^13^ In addition, in measurement with this technique, time, special device requirements, special equipment, and the use of contrast in invasive measurements are the limitations of this measurement.^11,12^

Conventionally, aortic elasticity measurements using echocardiography are based on diameter changes in the systole and diastole of M-mode slices of the ascending aorta.^17^ However, we hypothesized that these measurements would be unreliable in BAV due to changes in the geometric structure of the aortic root. Furthermore, because blood pressure is measured non-invasively from the periphery, it may not reflect aortic distensibility or stiffness parameters. Therefore, we aimed to evaluate the accuracy of PWV measurements using transthoracic echocardiography without additional equipment or software. Based on the principle that the distance traveled per unit of time is the path, we calculated the local PWV value by calculating the ratio of the distance between the measured locations between the ascending and descending aorta to the difference in blood travel time to the ascending and descending aortas. In our study, despite aortic dilatation in the BAV group, the elasticity parameters obtained using the conventional method did not differ between the groups. Findings similar to ours in one study support this hypothesis.^35^ In BAV, the axis of the fused segment is long, whereas the axis perpendicular to the fused segment is shorter. In type 1 BAV, the long axis of the aorta is parallel to the echocardiography probe, whereas in type 2, it is perpendicular to the probe (Figure 4). In our study, the ascending aortic diameter in the type 1 BAV group was higher than that in the type 2 BAV group, although the difference was not statistically significant, which further supports this hypothesis.

**Figure 4:** – Orientation of the probe relative to the aortic axis according to bicuspid aortic valve phenotypes. On the left, the long axis of the type 1 fused bicuspid aortic valve is perpendicular to the probe angle, whereas on the right, the short axis of the aorta in the type 2 fused bicuspid aortic valve is perpendicular to the probe diameter. LCC: left coronary cusp; RCC: right coronary cusp; NCC: noncoronary cusp.

In our study, the ventricular systolic and diastolic functions were normal in the BAV group. The BAV patients we evaluated were selected from those with normal or near-normal valve function. In clinical practice, pulse pressure increases when aortic elasticity is impaired. The normal blood pressure and pulse pressure values across the groups in our study may be related to the fact that impaired aortic elasticity has not yet caused ventricular or other organ dysfunction. However, long-term follow-up is necessary in this regard.

The intact elasticity of the aorta may not be reflected in the peripheral arterial systems. Therefore, assessing peripheral arterial stiffness may not indicate aortic elasticity. Studies have indicated that aortic stiffness is the first to be affected by arterial stiffness, with peripheral stiffness developing later.^29,30^

The mechanism of aortic dilatation in BAV may be impaired wall stress due to aortic valve insufficiency or turbulence occurring in aortic valve stenosis.^36,37^ The risk of aortic dilatation has also been reported to increase regardless of valve function.^38^ Aortic root dilatation has been reported to be between 15.1 and 39.1% in patients with isolated BAV.^39^ In the present study, the incidence of aortic root dilatation was 16%. Since patients in the BAV group in our study were selected with normal or near-normal valve function, evaluation of the incidence of aortic root dilatation will not yield accurate results. BAV is an independent risk factor for aortic dissection.^40^

Studies report that PWV is high or normal in BAV patients.^3, 41-45^ Increased PWV may impair aortic function in determining blood distribution.^26^This may lead to cardiovascular disease.^46^ PWV intraobserver and interobserver reliability alpha values were 0.92 and 0.84, respectively. The high intraobserver and interobserver values indicate the reliability and reproducibility of the measurement technique.^47^ In our study, the difference in the PWV mean and standard deviation values was 0.19 and 0.54 m/s between observers, respectively. It shows excellent agreement according to the ARTERY Society guideline.^48^Although our measurement method was different, we believe that these values may be significant. A limitation of our study is that we did not compare PWV measurements using sonographic methods with those obtained using echocardiography. We believe that the practicality of the measurement technique of PWV without the need for an extra apparatus, its reproducibility, and its high reliability will provide important information about the follow-up and prognosis of the patients.

### Limitations

The single center and small number of patients were the main limitations of this study. Another limitation is that the number of patients in the type 2 BAV group was lower than that in the type 1 BAV group. In PWV measurement, the distance between the ascending and descending aortas was measured intermittently using the current device. Measurement errors were another limitation of this study. The interobserver reliability alpha value was 0.78, which is the lowest value, and uninterrupted measurement of the inclined distance on the device can eliminate this disadvantage. Another limitation is that PWV is affected by the blood viscosity. Another limitation is the lack of a feasibility study of PWV measurement techniques. A limitation of our study is that we did not compare PWV measurements using sonographic methods with those obtained using echocardiography.

## Conclusions

The increase in aortic stiffness in patients with BAV was demonstrated by the PWV value with the same echocardiography device, without the need for an additional device. Although they are normal when evaluated with elasticity parameters derived from aortic diameters using echocardiography, aortic elasticity is reduced even in the early period when evaluated with PWV. Evaluation of patients with BAV with aortic elasticity parameters measured conventionally by echocardiography may yield incorrect results. We believe that PWV measurement with an echocardiography device, evaluation, and follow-up in patients with BAV can provide more useful information. In patients with BAV, even minimal aortic valve disease, particularly aortic regurgitation, may cause deterioration of aortic elasticity. We believe that, in the follow-up of patients with BAV, especially in the presence of aortic regurgitation, evaluation with PWV, in addition to the conventional method for evaluating aortic elasticity properties, will provide more accurate information. The high intraobserver and interobserver reliabilities of PWV measured using transthoracic echocardiography show that it is a clinically reproducible and reliable technique.
